# The Effects of Enzymes, Species, and Storage of Raw Material on Physicochemical Properties of Protein Hydrolysates from Whitefish Heads

**DOI:** 10.3390/md21110587

**Published:** 2023-11-10

**Authors:** Jannicke Fugledal Remme, Sigurd Korsnes, Stine Steen, Rachel Durand, Kristine Kvangarsnes, Janne Stangeland

**Affiliations:** 1 SINTEF Ålesund AS, Department of Fishery, Aquaculture and Process Technology, Borgundvegen 340, 6009 Aalesund, Norway; sigurd.korsnes@sintef.no (S.K.); stine.steen@sintef.no (S.S.); 2 SINTEF Ocean AS, Department of Fishery and New Biomarine Industry, Borgundvegen 340, 6009 Aalesund, Norway; rachel.durand@sintef.no; 3 Department of Biological Sciences Ålesund, Norwegian University of Science and Technology, Larsgårdsvegen 2, 6009 Ålesund, Norway; kristine.kvangarsnes@ntnu.no; 4 Møreforsking AS, Borgundvegen 340, 6009 Ålesund, Norway; janne.kristin.stangeland@moreforsking.no

**Keywords:** fish heads, whitefish, rest raw materials, enzymatic hydrolysis, protein hydrolysate, molecular weight distribution

## Abstract

The rest raw materials of whitefish have great potential for increased utilisation and value creation. Whitefish heads have a high protein content and should be considered a healthy protein source for the growing population’s demands for sustainable protein. In this study, the heads of four different species of whitefish were processed via enzymatic hydrolysis, namely cod (*Gadus morhua*), cusk (*Brosme bromse*), haddock (*Melanogrammus aeglefinus*), and saithe (*Pollachius virens*), using three commercially available enzymes. Trials were conducted after 0, 3, and 6 months of the frozen storage of heads. A proximate analysis, molecular weight distribution, and protein solubility were evaluated for each of the products. The results show that, although the enzymatic hydrolysis of rest raw materials from different species of whitefish yielded products of slightly different characteristics, this process is viable for the production of high-quality protein from cod, cusk, haddock, and saithe heads. Six months of frozen storage of heads had a minimal effect on the yield and proximate composition of hydrolysates.

## 1. Introduction

The sustainable utilisation of marine resources has become increasingly important in our attempt to prevent hunger and the climate crisis in a world with a growing population. Indeed, fish rest raw materials (RRMs), such as heads, viscera, backbones, or skin, contain valuable proteins, lipids, and mineral fractions [1]. However, the degree of utilisation of RRM highly depends on the fish species, fishing method, onboard handling, and storage. In Norway, for example, aquaculture and small pelagic industries utilise nearly 100% of their RRMs. On the other hand, only 56% of wild whitefish RRMs, mainly from the species cod (*Gadus morhua*), saithe (*Pollachius virens*), cusk (*Brosme brosme*), and haddock (*Melanogrammus aeglefinus*), are used [2]. Around 708,000 tons of whitefish were caught in Norway in 2021 [3], where 40–50% are regarded as edible consumer products (filets). Generally, whitefish heads make up around 20.2% of the total weight of cod, 15.3% of saithe, 18.7% of haddock, and 17.9% of tusk [4], and they represent above 110,000 tons of rest raw material in Norway [2]. Moreover, whitefish heads have a high potential for increased and better utilisation and value creation, such as the production of novel food ingredients, nutraceuticals, and other value-added products.

Norwegian whitefish production is divided into the following two main industries: the (1) coastal fleet and the (2) oceanic fleet. The coastal fleet delivers fresh, whole fish during winter using almost all the rest raw materials produced. On the other hand, the oceanic fleet delivers frozen-headed and gutted fish throughout the year (accounting for two-thirds of Norwegian whitefish production); it has the potential to increase the utilisation and value creation from RRM due to the lack of space required and high onboard production costs [5]. Traditionally, large volumes of fresh cod heads from the coastal fleet are air-dried and mainly exported to Africa [6]. However, due to market instability, decreased profitability, and an increased focus on sustainability and climate action, other uses of whitefish heads have been investigated [7,8].

Enzymatic hydrolysis can be used to recover proteins and lipids from the RRM. Enzymatic hydrolysis performances and hydrolysate quality mostly depend on the enzyme and its concentration, the reaction temperature, pH and time, the enzyme–substrate interactions, and the raw material’s quality. During the hydrolysis process, short-chain peptides are released, resulting in hydrolysates with higher solubility [9,10]. Protein solubility is one of the most important of the functional properties, and many functional properties, such as emulsification and foaming, are affected by the solubility [11]. In addition, protein solubility may impact the digestibility of the protein. High protein solubility can also contribute to improved texture and stability in food products. Remme et al. (2022) showed that both fresh and frozen cod heads, due to their low lipid content (4%) and their high protein content (14–15%), are a good source for the production of fish protein hydrolysates (FPHs) [7,8].

In order to improve access to RRMs, frozen storage allows a longer period between catch/production and processing. However, the freezing process of fish can lead to protein aggregation, denaturation, cross-linking, and the breakdown of polypeptide chains [12,13]. Such changes in the protein structure can reduce the nutritional value of the product (e.g., reduced digestibility), decrease the production yield of FPH, modify the organoleptic properties, and alter protein solubility and other functional properties [12]. It is, therefore, important that the heads can be frozen over a period of time without a loss of quality. Moreover, the Norwegian whitefish industry produces different species, such as cod, saithe, cusk, and haddock. Although they are all whitefish, it is important to know how their differences and similarities impact the enzymatic hydrolysis performance and the characterisation of the final product. This knowledge can be used to choose further forms of process and product development, choosing to either process whitefish heads together or as individual species.

With the prospect of developing a new protein industry based on whitefish heads, commercial and food-grade enzymes were used in these trials. Remme et al. (2022) [7,8] showed that a combination of papain and bromelain (1:1) gave the best yield for the hydrolysis of cod heads. This combination was, therefore, added for comparison reasons. As the coastal fleet delivers whitefish heads for a short period of time, including frozen heads from the oceanic fleet, it contributes to the increased sustainability of the fleet and attractive year-round workplaces onshore. The large-scale production of high-quality FPH from whitefish heads could provide an economically stable market for whitefish heads for both the coastal and oceanic fleets throughout the year. The main aim of this study is to evaluate the production of FPHs under different enzymatic hydrolysis conditions and assess the impacts of fish species and the frozen storage time on the end product’s quality.

## 2. Results and Discussion

All the hydrolysis trials produced two fractions: (1) fish protein hydrolysates (FPHs) and (2) sediments. The number of lipids in whitefish heads was insufficient to produce a separate fraction after hydrolysis, indicating that any lipids present were distributed between the FPH and sediment fractions. Indeed, in their trials, using a mixture of cod viscera and backbones, Slizyte et al. reported that a minimum of 6 g of lipid per 100 g of wet-weight raw material was required in order to produce a separate oil fraction [14]. The cod heads in the studies by Remme et al. (2022) [7,8] also had a lipid content (below 1%), which was insufficient to produce a separate lipid fraction.

### 2.1. Raw Material

The chemical composition of whitefish heads was determined, and the composition of cod heads was 15.4 ± 1.0% protein, 0.9 ± 0.1% lipid, 77.9 ± 1.3% water, and 6.7 ± 1.0% ash [7]. For comparison, a previous study [15] revealed values for minced cod head compositions of 11.3 ± 1.9% protein (*n* = 20), 3.6 ± 0.4% lipids (*n* = 10), 78.7 ± 1.3% water (*n* = 15), and 6.7 ± 1.1% ash (*n* = 15). The chemical composition of saithe heads was 17.3 ± 1.2% protein, 78.9 ± 1.4% water, and 5.8 ± 0.9% ash. The chemical composition of haddock heads was 17.1 ±1.0% protein, 79.1 ± 0.4% water, and 6.9 ± 0.5% ash. These results are in accordance with the results published by Økland and Kjerstad (2002) [16]. The chemical composition of cusk heads was 16.2 ± 1.2% protein, 74.9 ± 1.4% water, and 8.5 ± 1.3% ash.

### 2.2. Dry Matter

The liquid hydrolysates dry matter content (DM) ranged from 4.93 to 6.58% (Table 1), which is in accordance with the results from hydrolysis under similar conditions in previous studies of cod heads [7,8]. The cusk head protein hydrolysate (CuPH) had a significantly higher DM regardless of the enzyme and storage time compared to cod head protein hydrolysates (CPHs) and saithe head protein hydrolysates (SPHs), while haddock head hydrolysates (HPHs) had a significantly lower DM. Indeed, DM values were significantly affected by the species (*p* < 0.05) and enzyme (*p* < 0.05), while storage time was found to be non-significant (*p* = 0.209). 

### 2.3. Protein Yield

The protein yield (g protein/100 g raw material) ranged from 6.6 ± 0.7% to 8.0 ± 0.2% for cod, from 7.9 ± 0.2% to 9.7 ± 0.1% for cusk, from 6.3 ± 0.1% to 7.1 ± 0.1% for haddock, and from 6.8 ± 0.1% to 7.6 ± 0.3% for saithe (Table 1). The highest yield of 9.7 g protein/100 g of raw material was achieved for CuPH hydrolysed by Alcalase^®^ and stored for 3 months. The results showed a significant three-way interaction between the species, enzyme, and storage time (*p* < 0.05) on the protein yield. Further investigation of the two-way interaction between the species and enzyme showed that this interaction was only significant for raw material stored for 0 months. Furthermore, the main effect of this species was significant for all enzyme types, which indicated that a comparison of the hydrolysis performance across species would not provide more useful information. Two-way ANOVA within each species (Table 1) showed that there were no significant differences within the haddock and saithe groups and that, in addition, the enzyme had the only significant effect on the protein yield for both groups (*p* < 0.05 and *p* < 0.05). The two-way interaction between the enzyme and storage time was significant for CPH (*p* < 0.05), whereas for the HPHs, the simple main effect of the enzyme was significant (*p* < 0.05) for the protein yield. In brief, both the enzyme and storage time impacted the protein yield, but the main effect was mostly due to the different species used.

A comparison of saithe, cod, haddock, and cusk (one-way ANOVA with species) showed that cusk was significantly different from cod and saithe (*p* < 0.05), while the other three were significantly different from haddock with regard to protein yield. Hydrolysates produced from cusk heads had, on average, and regardless of the enzyme and storage time, a higher protein yield compared to cod, saithe, and haddock.

### 2.4. Chemical Composition of Dry Powder Hydrolysates

The proximate chemical composition of dried FPHs is shown in Table 2. High-quality hydrolysates for human consumption are characterised by a high content of digestible proteins and a low lipid and ash content.

Whitefish heads are generally low in lipids, which is reflected in the fat content of hydrolysates obtained in this study (Table 2). The average fat contents of CPHs and HPHs were 0.40 ± 0.09% and 0.44 ± 0.09% on the dry weight basis, respectively, indicating that neither the storage time nor the enzyme significantly affected the amount of fat in the hydrolysate. However, the fat contents of SPHs and CuPHs were, on average, 1.1 ± 1.7% and 0.8 ± 0.4%, respectively. The SPH after 3 months of storage had a far higher fat content (ranging from 0.3 to 7.0%, 2.4% on average) compared to the averages from 0 and 6 months of storage (0.4 ± 0.1 and 0.4 ± 0.1%, respectively), which could be a result of major differences in the raw material. Fraction separation via centrifugation did not result in any free oil phase production, which has also been observed in studies on cod head processing [15,17]. Lipid oxidation is of great concern to the food industry and consumers because it results in unpalatable flavours, unpleasant odours, dark colouring, and potentially toxic reaction products [18,19,20]. The Food and Agriculture Organization of the United Nations has issued a standard stipulating that the lipid content in FPHs used for human consumption must not exceed 0.5% (*w*/*w*) [21]. The use of commercial enzymes can produce hydrolysates with a low lipid content, whereas hydrolysis with only endogenous enzymes can fail to have the same positive effect on lipid concentrations [8]. Overall, the three commercial proteases used in this study showed a high efficiency in producing hydrolysates with low lipid contents compared to endogenous enzyme efficacies [8].

The ash contents in dried FPHs are shown in Table 2, and they differed significantly depending on the species (*p* < 0.05). The ash content ranged from 10.0 to 12.2% for CPHs, from 10.7% to 13.3% for SPHs, from 10.6% to 13.8% for HPHs, and from 8.1% to 9.0% for CuPHs. Previous studies have shown that FPHs from cod heads of the oceanic fleet (trawl and longline) have an ash content of 7.5%–8.2% [7], whereas FPHs from cod heads of the coastal fleet have an ash content of 9.6%–17.2% [8]. The ash content in FPHs from cod heads is high [7,8] compared to other species, for example, salmon (2.2%) [22]. A pairwise comparison showed that the ash content was significantly lower in CuPHs compared to the other hydrolysates, with an average of 8.4%, whereas HPHs had the highest ash content, with an average of 12.7%. A three-way ANOVA showed that there was a significant three-way interaction between the species, enzyme, and storage time with the ash content as the response (*p* < 0.05). All the simple main effects were also significant. A one-way ANOVA showed that there was no significant difference between the different groups of enzymes (*p* = 0.238), which was also the case for storage time (*p* = 0.063).

Proteins are one of the most valuable components in fish heads [23]. The obtained FPHs had a higher protein content compared to other studies, which reported the protein contents of different fish protein hydrolysates to be between 50% and 90% [24,25,26,27,28]. However, they were in accordance with previous results on cod heads [7,8]. The high protein content demonstrates their potential for use as protein ingredients for human consumption. However, the high ash content of the FPHs is a challenge for direct human consumption and should be considered for the industrial scale-up of RRM hydrolysis. The use of filtration membrane technologies (ultra- and nanofiltration) has been shown to be an economical, fast, and efficient process to reduce the salt contents in different hydrolysate sources [29,30]. 

### 2.5. Molecular Weight Distribution

**Figure 1:**
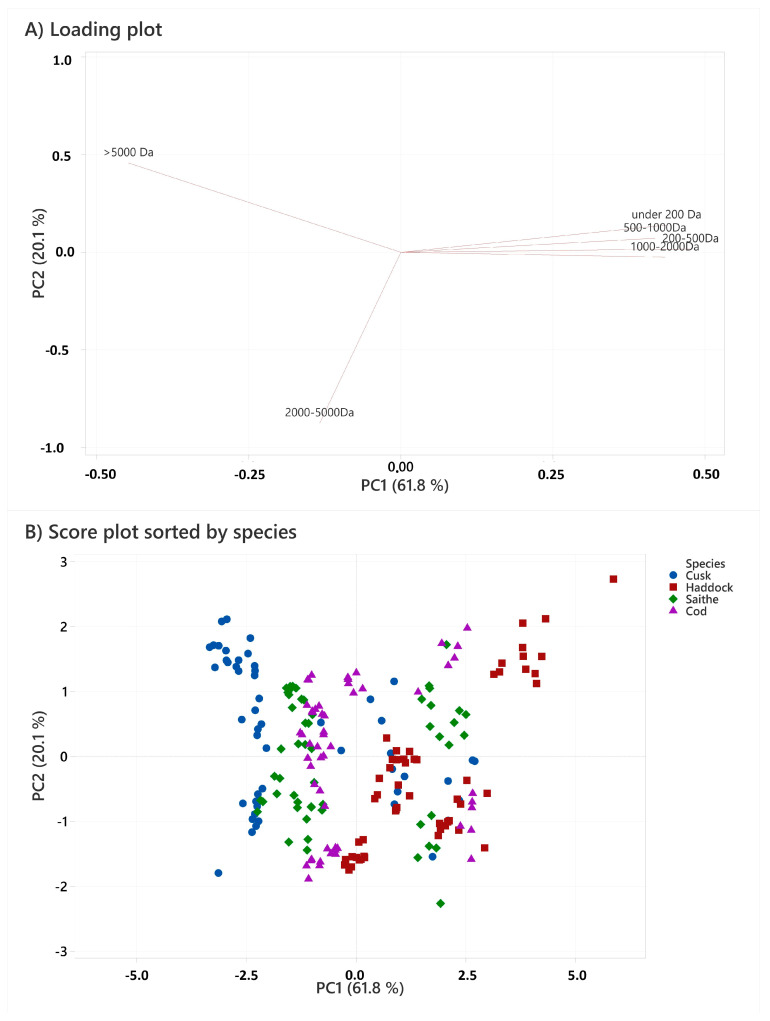

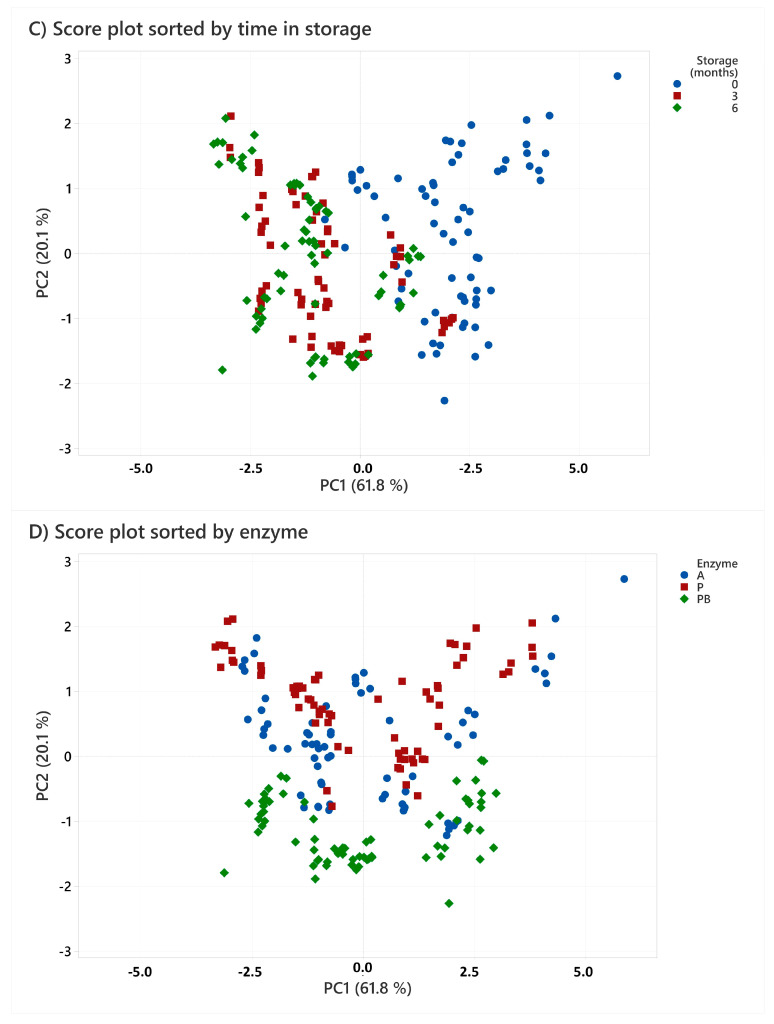
(**A**) PCA loading plot based on molecular weight distribution (Table 3). Variables close together with similar angles of origin are positively correlated, while variables that are opposed and with angles nearing 180° through the origin are negatively correlated. Variables that have a 90° angle between them through the origin are non-correlated. (**B**) PCA score plot sorted by fish species. (**C**) PCA score plot sorted by time in storage. (**D**) PCA score plot sorted by enzyme, showing the similarities and differences between the FPH molecular weight distributions. Objects in the score plot that are close together are similar, whereas objects that are further apart are increasingly different with regard to the loadings, explaining the given components.

The peptide molecular weight is of special interest for FPH sensory characteristics, such as bitterness [31,32,33,34,35,36], as well as functional properties, like emulsion, solubility capacity [23,37,38], and bioactivities [25,39,40,41]. The formation of bitter taste is mainly ascribed to small peptides of less than 1000 Da or up to 8–10 amino acids [31,42]. In hydrolysing whitefish heads, the aim of this study was to produce as many peptides in the range between 5000 Da and 500 Da as possible. Previous studies by Remme et al. (2022) [7,8] have shown that FPHs that consist of peptides in this range are easy to solve in water and are nearly neutral in taste. The molecular weight (MW) distribution of the produced FPHs has been sorted into three main categories: >5000 Da, 2000 to 5000 Da, and <2000 Da (Table 3). A principal component analysis (PCA) was used to evaluate the MW distribution of the hydrolysates, and the first two components explained 61.8% and 20.1% of the variation, respectively. Figure 1 contains three identical score plots that show the similarities and dissimilarities between the hydrolysates, with three different grouping categories for colour coding.

The results show that hydrolysates produced with raw materials and stored for 0 months contain more peptides above 2000 Da compared to FPHs from raw materials that were stored for 3 and 6 months. In comparison to the samples grouped by storage time, there was a separation between the samples stored for 0 months and those stored for 3 and 6 months. The loadings plot showed that the variation in lower MW (<2000) could mostly be explained by PC1, while the variation in MW > 5000 Da was equally explained by PC1 and PC2, and the variations in MW 2000–5000 Da were almost entirely explained by PC2. There are no clear groupings in the score plot (Figure 1) when highlighting species, but most CuPHs tend to be less associated with lower MW variables and more so with higher MW variables. When studying frozen heads from the oceanic fleet, the number of peptides below 5000 Da (74.3%) was almost similar [7,8]. These results are in accordance with FPHs made from heads that were stored for 3 and 6 months. However, in the FPHs produced with heads from the oceanic fleet, 56.9% of the peptides were below 2000 Da, and 23.5% peptides were below 1000 Da. These results are in accordance with the fresher raw materials in this study.

Also, hydrolysates produced using PB enzymes showed a higher content of MW peptides ranging between 2000 and 5000 Da compared to the other enzyme combinations. This is in accordance with studies by Remme et al. (2022) on cod head hydrolysis with PBs, which reported that 78.3% of peptides were below 5000 D, 39.4% were below 2000 Da, and 19.8% were below 1000 Da [7,8,15].

### 2.6. Protein Solubility of FPH

The solubility of proteins is shown in Table 4. A one-way Welch ANOVA was conducted to determine if the solubility of proteins was different in FPHs made from heads stored at different time ranges. Regarding the water solubility of proteins in FPHs made from cod, there was no difference in the solubility of FPHs made from heads stored for different periods of time (0, 3, or 6 months). For haddock and saithe, there were no significant differences in the solubility of proteins in FPHs made from heads stored for 0 and 3 months. Although there were significant differences (*p* < 0.05) in the soluble proteins of FPHs made from tusk heads stored for 0 and 3 months, these differences were small. In the case of FPHs made from the heads of haddock, saithe, and tusk and stored for 6 months, this protein was less soluble compared to the protein in FPHs made from heads stored for 0 and 3 months. During frozen storage, both structural and chemical properties may be altered [43], which can affect both the denaturation and solubility of proteins. A lower solubility during frozen storage has been observed in studies where mackerel have been frozen [44], which is in accordance with Geirsdottir et al. (2007) [41], who found that the solubility in herring muscle decreased during frozen storage for 6 months, where the solubility was about 10% lower after 6 months of frozen storage compared to the beginning (*p* < 0.05). The solubility of proteins in FPHs has been linked to protein oxidation in raw materials [25]. The minced heads of trout were exposed to pro-oxidants with protein oxidation as a consequence. FPHs made of the most oxidated raw material had the lowest content of soluble proteins. It is likely that changes in the raw material, when stored for 6 months, cause the lower solubility of proteins in FPHs. The described differences in solubility between different storage times of heads were independent of which enzyme was used. However, there were some interesting differences in the solubility regarding which enzyme was used when the FPH was made of heads stored for six months. For FPHs made of saith and tusk heads stored for 6 months, the water solubility of proteins was significantly (*p* < 0.05) lower in FPHs prepared with Alcalase compared to Protamex and Bromelain/Papain. A lower solubility was also observed in FPHs from the heads of haddock stored for 6 months, but this difference was not significant. Other studies have suggested that Alcalase exhibits higher hydrolytic activity compared to Protamex when protein hydrolysates are prepared from tuna heads [45]. However, there were no significant differences in the solubility of FPHs made from different enzymes with heads stored for 0 or 3 months. Therefore, changes that occurred during the storage of heads might affect the effectiveness of Alcalase. Therefore, it is of interest to include the oxidation parameters and other quality parameters of raw material in further studies. At the same time, a lower degree of solubility should be reflected in a smaller proportion of smaller peptide sizes, which we did not find in our experiments. Therefore, the lower solubility in FPHs from heads stored for 6 months made with Alcalase must originate from something other than lower peptide sizes.

## 3. Materials and Methods

### 3.1. Raw Material

The heads of cod (*Gadus morhua*), saithe (*Pollachius virens*), cusk (*Brosme brosme*), and haddock (*Melanogrammus aeglefinus*) were provided via longline fishing vessels. All species were captured in zone FAO 27. All fish were captured in 2021, with saithe and haddock in March, cod in May, and cusk in August. All species were beheaded and gutted on board. Heads were collected in 25 kg batches, frozen (−20 °C), and transported to SINTEF Ocean (Trondheim, Norway), where they were kept frozen for 0, 3, and 6 months at −18 °C. Prior to hydrolysis trials, frozen heads (*n* = 10) were thawed in a refrigerated room at 4 °C for 18 h before being minced (*n* = 3) in an AE200 mincer (Hobart Corporation, Troy, OH, USA) with 10 mm diameter holes.

### 3.2. Chemicals and Enzymes

The enzymes used were Alcalase^®^ (2.4 U/g) and Protamex^®^ (1.5 AU-N/g), provided by Novozymes (Bagsvaerd, Denmark), and Papain (Performase ^®^GSM80) and Bromelain (2400 GDU/g) was provided by Enzybel International S.A. (Villers-le-Bouillet, Waterloo, Belgium). Papain and Bromelain were used in a 1:1 (*w*/*w*) combination. The enzymes used in this study comply with the recommended purity specifications for food-grade enzymes issued by the joint FAO/WHO Expert Committee on Food Additives (JECFA) and the Food Chemicals Codex (FCC). Methanol, chloroform, hexane, and formaldehyde were used for chemical analysis and obtained from Merck (Darmstad, Germany). Folin reagent and BSA standard were obtained from Sigma Aldrich. Cytochrome C, aprotinin, insulin A, Leu-enkephalin, Val-Tyr-Val, and Gly-Tyr were used as standards for the determination of molecular weight distributions, which were all obtained from Sigma-Aldrich (St. Louis, MO, USA). All chemicals used were of reagent grade.

### 3.3. Enzymatic Hydrolysis

Hydrolysis was performed in a closed, 4 L glass reactor with an RZR 2021 electrical impeller (Heidolph Instruments, Schwabach, Germany). Minced heads (1 kg) were mixed with pre-heated water (50 °C) in a ratio of 1:1 (*w*/*w*) based on the factors listed in Table 5. After the mixture reached 50 °C, hydrolysis was started by adding 0.1% of the enzyme (% *w*/*w* of raw material) to the mixture. After 60 min, the enzymes were inactivated at 90 °C for 10 min before centrifugation at 2250× *g* for 15 min using a Heraeus Multifuge X3R (ThermoFisher Scientific, Waltham, MA, USA). The water-soluble fraction was separated from the insoluble fraction via manual decantation. All hydrolysis trials were performed in duplicate.

### 3.4. Chemical Characterisation

The moisture in raw material, wet, and freeze-dried hydrolysates was determined gravimetrically after drying at 105 °C for 24 h. The ash content was estimated using the AOAC Official Method 942.05. Total nitrogen (N) was determined using a CHNSO analyser (ECS4010, Costech Analytical Technologies, Valencia, CA, USA). Crude protein was estimated by multiplying the total N by a factor of 6.25 [46]. Lipid content was determined gravimetrically after extraction using the Bligh and Dyer method [47]. All samples were analysed in triplicate.

### 3.5. Molecular Weight Distribution

Freeze-dried FPH was diluted with Milli-Q (MQ) water to a concentration of 10 mg/mL. Then, 100 µL of the diluted FPH solution was further diluted with 900 µL of 10% acetonitrile in MQ water in an HPLC vial. Analysis was performed on an AQUITY UPLC H-Class PLUS System (Waters Corporation, Milford, MA, USA) with an AQUITY BEH125 SEC 1.7u 4.6 mm × 150 mm column (Waters) and an AQUITY UPLC PDA Detector (Waters Corporation, Milford, MA, USA) set to 220 nm. Runs were isocratic, and a 100 mM phosphate buffer (pH 6.8) was used as the mobile phase with 0.4 mL/min of flow rate, an injection volume of 2 µL, and a total run time of 10 min. The column temperature was set to 30 °C for analysis. Cytochrome C (12327 Da), aprotinin (6512 Da), insulin A (2531 Da), Leu-enkephalin (555.6 Da), Val-Tyr-Val (379.5 Da), and Gly-Tyr (238.2 Da) were used as standards. Chromatograms were manually integrated and separated into intervals of <0.2, 0.2–0.5, 0.5–1, 1–2, 2–5, and >5 kDa, expressed as percentages of the total area. All samples were analysed in triplicate.

### 3.6. Protein Solubility

Protein extracts were prepared by dissolving 0.1 g of each FPH sample in 10 mL of distilled water. The solutions were homogenised and centrifuged. Water-soluble proteins were determined using the Lowry method [48] and measured in triplicates. Bovine serum albumin (BSA) was used as a standard. The absorbance of the incubated standards and samples was determined using a GENESYS 10 UV-VIS Spectrophotometer (Thermo Fisher Scientific Inc., USA) at a wavelength of 750 nm. The protein solubility was calculated from the following formula:

$$Solubility\;\left(\%\right)=\frac{Ps}{Pt}\times 100$$

where *Ps* is soluble proteins and *Pt* is total protein content in the sample.

### 3.7. Statistical Analysis

All statistical analyses were performed using Minitab 20.3 and STATA (v.18.0). Each test was conducted at a 95% confidence level. A three-way analysis of variance (ANOVA) was conducted to determine how the factor and its interactions affected the response variables. For cases where a two- or three-way interaction was significant, the interaction was broken into either n (number levels per factor) one-way ANOVAs, one per level of the second factor, or n two-way ANOVAs, one per level of the third factor. This exercise was conducted to determine at which conditions the interactions were significant. Tukey’s pairwise comparison was used as a post hoc comparison of the mean. One-way ANOVA with Tukey’s HSD as a post hoc test was used to determine significant differences in the solubility of heads with different storage times or with the use of different enzymes. When data did not meet the homogeneity of variances requirement for one-way ANOVA, a Welch ANOVA was applied, with Games and Howell as the post hoc test. Principal component analysis (PCA) was used to compare molecular weight distributions. The variables were standardised by mean centring and dividing by the standard deviation of each entry to achieve a mean of 0 and a standard deviation of 1.

## 4. Conclusions

Protein hydrolysates from cod, saithe, and haddock heads, produced under comparable conditions, have similar protein yields, chemical compositions, and MW distributions. This indicates that these heads can be mixed for the production of high-quality protein hydrolysates. Cusk, on the other hand, differs in both its chemical composition and MW distribution, and it should not be mixed with the other heads. The heads can be stored frozen without changes in their yield or chemical composition. However, the MW distribution can vary as a factor of storage. The results also indicate that solubility decreases with storage.

## Figures and Tables

**Table 1:** Dry matter (% of wet weight) and protein yield (g protein/100 g raw material) in hydrolysates from cod, saithe, haddock, and cusk after zero, three, and six months of frozen storage using three different enzymes (PB = Papain and Bromelain (1:1 *w*/*w*), P = Protamex^®^, and A = Alcalase^®^).

Species	Storage	Enzyme	Dry Matter			Protein Yield		
	(Months)	(Type)	(% of Wet Weight)			(g/100 g Raw Material)		
Cod	0	A	5.19	±	0.02 d	6.61	±	0.10 d
		P	5.79	±	0.02 bc	7.15	±	0.09 bcd
		PB	6.27	±	0.03 a	8.03	±	0.20 a
	3	A	5.60	±	0.01 c	7.49	±	0.00 abc
		P	5.62	±	0.04 c	7.24	±	0.09 abcd
		PB	5.93	±	0.09 b	7.82	±	0.29 ab
	6	A	5.80	±	0.00 bc	7.28	±	0.01 abcd
		P	5.65	±	0.03 c	6.56	±	0.12 d
		PB	5.96	±	0.01 b	6.92	±	0.12 cd
Cusk	0	A	6.58	±	0.07 a	8.26	±	0.03 bc
		P	6.39	±	0.18 a	7.86	±	0.21 c
		PB	6.54	±	0.14 a	8.01	±	0.50 bc
	3	A	6.58	±	0.02 a	9.66	±	0.07 a
		P	6.67	±	0.01 a	9.40	±	0.03 a
		PB	6.67	±	0.07 a	9.37	±	0.11 a
	6	A	6.34	±	0.00 a	8.98	±	0.10 ab
		P	6.34	±	0.10 a	8.77	±	0.13 abc
		PB	6.57	±	0.07 a	8.75	±	0.01 abc
Haddock	0	A	5.30	±	0.02 a	6.79	±	0.06 a
		P	5.35	±	0.17 a	6.70	±	0.20 a
		PB	5.18	±	0.02 a	6.46	±	0.06 a
	3	A	5.33	±	0.04 a	7.10	±	0.05 a
		P	4.93	±	0.02 a	6.40	±	0.08 a
		PB	5.18	±	0.06 a	6.48	±	0.32 a
	6	A	5.26	±	0.04 a	6.63	±	0.02 a
		P	5.08	±	0.08 a	6.30	±	0.12 a
		PB	5.13	±	0.11 a	6.37	±	0.26 a
Saithe	0	A	5.65	±	0.01 ab	7.37	±	0.01 a
		P	5.68	±	0.06 ab	7.19	±	0.02 a
		PB	5.70	±	0.06 ab	7.10	±	0.04 a
	3	A	5.88	±	0.19 a	7.58	±	0.17 a
		P	5.42	±	0.04 b	6.94	±	0.21 a
		PB	5.81	±	0.03 ab	7.56	±	0.30 a
	6	A	5.52	±	0.02 ab	7.18	±	0.07 a
		P	5.41	±	0.02 b	6.80	±	0.09 a
		PB	5.55	±	0.02 ab	7.24	±	0.06 a

Values that do not share letters in the same column within the same species are significantly different.

**Table 2:** Chemical composition of freeze-dried FPHs produced from the heads of cod, saithe, haddock, and cusk after zero, three, and six months of frozen storage using three different enzymes (PB = Papain and Bromelain (1:1 *w*/*w*), P = Protamex^®^, and A = Alcalase^®^). Values are given as mean ± standard deviation (*n* = 2). Values for ash, fat, and protein are given on a dry matter basis.

Species	Storage (Months)	Enzyme (Type)	Dry Matter (%)	Ash (%)	Fat (%)	Protein (%)
Cod	0	PB	96.80 ± 1.61	10.00 ± 0.06	0.50 ± 0.02	91.76 ± 0.89
		P	98.21 ± 0.22	11.74 ± 0.12	0.40 ± 0.01	88.58 ± 0.21
		A	97.63 ± 0.27	10.43 ± 0.39	0.32 ± 0.01	89.05 ± 0.07
	3	PB	97.34 ± 0.12	11.24 ± 0.16	0.48 ± 0.04	91.78 ± 0.49
		P	96.99 ± 0.16	12.18 ± 0.24	0.48 ± 0.04	90.57 ± 0.22
		A	96.67 ± 0.02	11.71 ± 0.01	0.35 ± 0.04	90.08 ± 0.24
	6	PB	98.39 ± 0.03	11.86 ± 0.31	0.29 ± 0.02	80.74 ± 0.77
		P	98.11 ± 0.30	11.31 ± 0.20	0.47 ± 0.06	83.35 ± 0.37
		A	97.73 ± 0.33	10.75 ± 0.17	0.32 ± 0.01	83.46 ± 0.11
Cusk	0	PB	98.06 ± 0.15	8.58 ± 0.25	0.55 ± 0.04	88.21 ± 1.37
		P	98.12 ± 0.02	8.80 ± 0.13	1.42 ± 0.21	88.01 ± 0.40
		A	98.36 ± 0.17	8.07 ± 0.24	0.90 ± 0.36	89.01 ± 0.81
	3	PB	99.74 ± 0.06	8.96 ± 0.04	0.41 ± 0.03	97.62 ± 0.88
		P	99.63 ± 0.17	8.96 ± 0.05	1.17 ± 0.26	98.27 ± 0.14
		A	99.57 ± 0.06	8.43 ± 0.12	0.45 ± 0.14	98.09 ± 0.51
	6	PB	98.53 ± 0.11	8.18 ± 0.16	0.52 ± 0.06	94.57 ± 0.34
		P	98.78 ± 0.19	9.02 ± 0.32	0.80 ± 0.02	94.19 ± 0.16
		A	98.51 ± 0.12	8.47 ± 0.05	0.62 ± 0.02	94.34 ± 0.28
Haddock	0	PB	98.05 ± 0.09	11.09 ± 0.08	0.41 ± 0.05	88.80 ± 0.18
		P	98.54 ± 0.17	11.31 ± 0.25	0.40 ± 0.05	88.32 ± 0.56
		A	98.63 ± 0.00	10.58 ± 0.01	0.34 ± 0.10	88.05 ± 0.19
	3	PB	98.27 ± 0.03	13.52 ± 0.02	0.35 ± 0.02	86.46 ± 2.91
		P	99.24 ± 0.21	14.51 ± 0.09	0.52 ± 0.10	86.99 ± 0.99
		A	99.02 ± 0.02	13.02 ± 0.00	0.45 ± 0.04	87.51 ± 0.47
	6	PB	97.55 ± 0.29	13.84 ± 0.79	0.41 ± 0.03	84.35 ± 0.33
		P	97.91 ± 0.13	13.47 ± 1.54	0.55 ± 0.03	84.09 ± 0.15
		A	98.38 ± 0.19	13.06 ± 0.08	0.51 ± 0.01	85.65 ± 0.25
Saithe	0	PB	99.54 ± 0.13	11.12 ± 0.23	0.30 ± 0.05	87.39 ± 0.62
		P	99.66 ± 0.02	11.33 ± 0.46	0.39 ± 0.02	88.10 ± 0.48
		A	99.47 ± 0.17	10.74 ± 0.05	0.36 ± 0.00	87.70 ± 0.05
	3	PB	98.29 ± 0.71	11.86 ± 0.17	1.27 ± 0.16	90.19 ± 0.82
		P	99.23 ± 0.34	13.27 ± 0.13	2.38 ± 1.05	87.85 ± 1.29
		A	98.20 ± 0.97	11.68 ± 0.12	3.73 ± 3.39	88.53 ± 3.52
	6	PB	98.53 ± 0.16	10.89 ± 0.11	0.32 ± 0.01	89.25 ± 0.03
		P	98.81 ± 0.05	12.49 ± 0.09	0.55 ± 0.05	87.11 ± 0.04
		A	98.77 ± 0.08	11.20 ± 0.40	0.28 ± 0.03	87.54 ± 0.20

**Table 3:** Molecular weight distribution (%) of freeze-dried FPHs produced from the heads of cod, saithe, haddock, and cusk after zero, three, and six months of frozen storage using three different enzymes (PB = Papain and Bromelain, P = Protamex^®^, and A = Alcalase^®^). Values are given as the mean ± standard deviation (*n* = 3).

Species	Storage Time	Enzyme	>5000 Da			2000–5000 Da			1000–2000 Da			500–1000 Da			200–500 Da			<200 Da		
Cod	0	A	30.1	±	1.0	28.1	±	0.8	18.2	±	1.8	11.7	±	0.2	6.8	±	0.4	5.0	±	0.6
		P	21.3	±	1.4	23.6	±	1.9	26.2	±	2.9	13.8	±	0.5	8.1	±	0.3	6.9	±	1.9
		PB	6.7	±	1.8	35.5	±	2.3	29.0	±	0.7	15.3	±	0.1	8.6	±	1.6	4.9	±	1.5
	3	A	29.6	±	1.6	33.1	±	2.4	16.1	±	1.9	11.1	±	1.1	7.6	±	0.1	2.4	±	0.1
		P	30.4	±	4.8	31.8	±	4.7	17.4	±	1.3	11.0	±	0.9	6.9	±	0.7	2.5	±	0.1
		PB	19.7	±	0.5	40.8	±	0.4	19.6	±	2.0	11.0	±	1.5	6.9	±	0.2	1.9	±	0.1
	6	A	29.9	±	1.6	32.9	±	1.0	20.8	±	0.5	7.1	±	0.3	6.8	±	0.5	2.5	±	0.2
		P	31.4	±	0.8	29.4	±	0.2	22.9	±	0.2	7.5	±	0.5	6.5	±	0.8	2.3	±	0.2
		PB	20.5	±	0.5	41.9	±	0.6	23.0	±	0.4	6.6	±	0.6	5.8	±	0.9	2.3	±	0.2
Cusk	0	A	18.6	±	3.5	33.3	±	2.6	21.9	±	3.8	15.6	±	1.8	8.3	±	0.6	2.3	±	0.6
		P	24.5	±	3.5	30.6	±	2.8	19.2	±	0.9	16.4	±	1.2	7.3	±	1.9	2.0	±	1.4
		PB	9.8	±	1.2	33.2	±	3.7	27.6	±	3.4	17.9	±	1.3	9.3	±	0.5	2.2	±	0.3
	3	A	36.4	±	1.6	32.1	±	1.4	17.0	±	0.9	8.7	±	0.6	4.6	±	0.1	1.3	±	0.1
		P	43.1	±	2.9	26.9	±	1.4	16.8	±	1.0	7.9	±	0.7	4.1	±	0.3	1.1	±	0.4
		PB	30.9	±	0.5	38.0	±	0.8	18.2	±	0.5	7.5	±	0.2	4.3	±	0.1	1.0	±	0.1
	6	A	42.8	±	1.7	28.5	±	2.4	13.1	±	1.2	9.4	±	0.7	4.9	±	0.1	1.3	±	0.1
		P	46.5	±	1.4	26.9	±	1.2	14.7	±	0.4	6.8	±	0.5	4.1	±	0.3	1.0	±	0.3
		PB	30.2	±	1.1	40.5	±	2.4	17.6	±	2.5	6.7	±	1.7	4.2	±	0.3	0.9	±	0.2
Haddock	0	A	12.9	±	2.1	21.4	±	4.5	27.5	±	5.5	16.0	±	1.0	14.0	±	0.3	8.2	±	1.5
		P	15.9	±	0.8	22.3	±	1.7	27.9	±	1.9	14.5	±	2.3	12.8	±	0.6	6.8	±	0.9
		PB	8.9	±	2.3	36.2	±	2.2	23.2	±	2.1	14.6	±	1.2	9.7	±	0.7	7.2	±	1.4
	3	A	10.1	±	0.3	37.4	±	0.5	24.2	±	0.8	13.6	±	1.0	9.1	±	0.1	5.6	±	0.1
		P	20.4	±	1.4	32.9	±	1.2	22.4	±	0.8	10.5	±	0.5	8.8	±	0.3	4.9	±	0.2
		PB	17.6	±	0.7	41.2	±	0.8	18.7	±	1.2	10.3	±	1.1	8.5	±	0.2	3.8	±	0.2
	6	A	18.4	±	1.9	36.3	±	0.6	21.3	±	1.0	10.7	±	0.4	8.9	±	0.5	4.4	±	0.4
		P	18.6	±	1.2	33.3	±	1.3	22.0	±	0.3	11.0	±	0.2	9.4	±	0.3	5.6	±	0.1
		PB	18.2	±	0.6	42.0	±	0.5	18.5	±	0.2	8.7	±	0.3	9.1	±	0.7	3.4	±	0.2
Saithe	0	A	15.4	±	1.0	28.3	±	1.5	28.3	±	1.8	13.9	±	0.2	9.4	±	0.1	4.7	±	0.5
		P	21.2	±	1.3	26.8	±	2.3	23.2	±	1.6	14.0	±	0.5	9.5	±	0.6	5.3	±	0.7
		PB	9.2	±	2.8	38.1	±	2.4	28.2	±	1.0	12.4	±	0.7	8.7	±	0.8	3.4	±	0.4
	3	A	25.8	±	1.7	37.8	±	0.7	17.5	±	1.4	10.2	±	1.0	5.8	±	0.2	2.8	±	0.5
		P	33.7	±	1.9	30.1	±	0.7	18.0	±	0.6	10.3	±	0.5	5.4	±	0.3	2.5	±	0.2
		PB	24.8	±	1.8	40.3	±	1.5	16.3	±	0.5	10.4	±	0.5	5.9	±	0.3	2.2	±	0.1
	6	A	30.3	±	2.5	33.7	±	2.3	18.0	±	1.8	9.1	±	1.7	6.5	±	0.6	2.2	±	0.3
		P	35.7	±	0.9	28.8	±	0.4	18.5	±	1.5	8.6	±	1.6	6.0	±	0.2	2.4	±	0.2
		PB	31.0	±	0.7	37.8	±	1.7	16.0	±	3.3	7.8	±	1.3	5.7	±	0.2	1.7	±	0.2

**Table 4:** Protein solubility (g/100 g hydrolysate) in FPHs. Values are given as the mean ± standard deviation; *n* = 6, Table 1.

Species	Enzyme	Storage Time (Months)		
		0	3	6
Cod	PB	100 ± 0.1 a,A	99.5 ± 0.9 a,AB	100 ± 0.1 a,A
	P	100 ± 0.1 a,A	100 ± 0.1 a,B	99.8 ± 0.6 a,A
	A	100 ± 0.1 a,A	97.1 ± 2.7 a,A	99.9 ± 0.2 a,A
Haddock	PB	99.4 ± 1.0 a,A	99.5 ± 1.3 a,A	95.1 ± 1.0 b,A
	P	99.9 ± 0.2 a,A	100 ± 0.1 a,A	95.9 ± 2.3 b,A
	A	99.6 ± 0.9 a,A	99.6 ± 0.6 a,A	93.4 ± 1.7 b,A
Saithe	PB	99.7 ± 0.7 a,A	99.9 ± 0.2 a,A	94.4 ± 0.8 b,A
	P	99.7 ± 0.7 a,A	99.8 ± 0.5 a,A	94.7 ± 0.9 b,A
	A	100 ± 0.1 a,A	100 ± 0.1 a,A	89.6 ± 2.2 b,B
Tusk	PB	100 ± 0.1 a,A	97.3 ± 2.7 b,A	87.1 ± 1.2 c,A
	P	99.0 ± 0.5 a,A	100 ± 0.1 b,A	85.9 ± 0.6 c,A
	A	97.8 ± 2.0 a,B	98.7 ± 1.4 a,A	84.1 ± 4.0 b,B

Values with different small letters in a row are significantly different (*p* < 0.05). Values with different capital letters in a column within the same species and the same storage time are significantly different (*p* < 0.05).

**Table 5:** Factors and their levels for hydrolysis trials.

Species	Enzymes	Storage Times
Cod	Alcalase	0 months
Cusk	Papain and Bromelain	3 months
Haddock	Protamex	6 months
Saithe		

## Data Availability

The data presented in this study are available on request from the corresponding author. The data are not publicly available due to privacy.
